# Preventing zoonotic spillover through regulatory frameworks governing wildlife trade: A scoping review

**DOI:** 10.1371/journal.pone.0312012

**Published:** 2025-01-06

**Authors:** Raphael Aguiar, Ryan Gray, Eduardo Gallo-Cajiao, Arne Ruckert, Chloe Clifford Astbury, Ronald Labonté, Peter Tsasis, A. M. Viens, Mary Wiktorowicz

**Affiliations:** 1 School of Health Policy and Management, York University, Toronto, Ontario, Canada; 2 Dahdaleh Institute for Global Health Research, York University, Toronto, Ontario, Canada; 3 School of Global Health, York University, Toronto, Ontario, Canada; 4 Department of Human Dimensions of Natural Resources, Colorado State University, Fort Collins, Colorado, United States of America; 5 AMR Policy Accelerator, York University, Toronto, Ontario, Canada; 6 Global Strategy Lab, York University, Toronto, Ontario, Canada; 7 School of Epidemiology and Public Health, University of Ottawa, Ottawa, Ontario, Canada; National Veterinary Research Institute, NIGERIA

## Abstract

Wildlife trade can create adverse impacts for biodiversity and human health globally, including increased risks for zoonotic spillover that can lead to pandemics. Institutional responses to zoonotic threats posed by wildlife trade are diverse; understanding regulations governing wildlife trade is an important step for effective zoonotic spillover prevention measures. In this review, we focused on peer-reviewed studies and grey literature conducted on regulatory approaches that govern domestic and international wildlife trade in order to assess the role of local, national and global-level institutions in the prevention of zoonotic spillover and infection transmission between humans. The five-stage scoping review protocol described by Arksey and O’Malley to map key concepts and main sources and types of evidence available was followed to understand and analyze empirical evidence from peer-reviewed studies and grey literature conducted on regulatory approaches that govern domestic and international wildlife. Sources were included if they discuss at least one of three points: regulatory approaches governing the wild animal trade, including wild animal markets, traditional medicine or exotic pets; regulatory approaches governing importation of wild animals and the international wildlife supply chain; or the role of local, national, and global-level institutions in regulating wild animal trade for food, traditional medicine or exotic pets. A total of 1598 sources were retrieved, from which 32 sources were included in the final review (30 studies + 2 grey literature reports). Based on published literature, regulations governing wildlife trade are inconsistent within and between countries. Organizations regulating wildlife trade may have competing interests, which can lead to fragmentation and a lack in coordination and oversight. National compliance with international regulations can be an issue. Reducing the probability of spillover events in wildlife trade is key to prevent future pandemics. Our results indicate a need for enhanced regulatory harmonization within and between national and supranational regulations. Coordination and collaboration for prevention of zoonotic infection and spillover may be enhanced through future research focused on the effectiveness of timely Information sharing and global- and national- level harmonization of wildlife trade regulations.

## Introduction

With the advent of the COVID-19 pandemic, the public health impacts of wildlife trade are increasingly being studied [1, 2], including recent scholarship on zoonotic linkages of emerging and re-emerging infectious diseases [3]. Infectious disease outbreaks, epidemics, and pandemics have become more frequent, with SARS, influenza, Ebola, Zika, dengue, and plague outbreaks all occurring within about a 15-year period [4]. Approximately 75% of emerging and re-emerging infectious diseases are of zoonotic origin [5]; while the origin of SARS-CoV-2 is not definitively known, the zoonotic hypothesis gained much support, with bats believed to be the host and pangolins or racoon dogs as potential intermediate hosts [6–8]. Research also shows that early COVID-19 patients resided significantly closer to the to the Huanan Seafood Wholesale Market in Wuhan, suggesting the pivotal role of wildlife trade in spillover events and of the market in Wuhan as the source or spreader event that led to the COVID -19 pandemic [9].

Wildlife trade includes trade of wild animals for meat, traditional medicine products and exotic pets, and it can be defined as the domestic and international economic exchange of wildlife whose purpose includes consumption by humans, comprising full supply chains, from harvest (which may or may not entail killing) to points of sale to consumers, including markets and restaurants [10]. In economic terms, the global trade in wildlife has increased 500% in value since 2005, and 2000% since the 1980s [11]. From 1997 to 2016, the legal trade was worth between US$2.9 to 4.4 trillion [12]. The illegal wildlife trade, on the other hand, is estimated to involve US$7–23 billion each year [12]. The demand for wildlife products for use in traditional medicines are worth US$60 billion annually, and expected to increase to US$123 billion in China alone by 2023 [13]. Wildlife trade provides many opportunities for infectious diseases to be transmitted from animals to human handlers (i.e. zoonoses) by increasing the human-animal-environment interface [14]. The important risks of the legal and illegal wildlife trade for public health cannot be ignored.

Regulatory approaches addressing threats posed by zoonotic disease include wildlife trade bans that carry significant cultural, development, economic, and health implications [15–17]. Targeted mitigation approaches for wildlife markets have included temporary closing [18, 19], improved market biosecurity measures [20], bans of trade for specific classes of wildlife [21], and specific interventions related to supply, transport, sale, consumption, and environmental critical control points [22].

Furthermore, live-animal markets, wildlife markets and wet markets are often conflated, obscuring important differences in risk management approaches for these different market types, potentially leading to negative consequences such as xenophobic discrimination [16, 23]. Despite the fact that the Huanan market contained a wet, live-animal, and wildlife market, each of these markets have different characteristics. Live-animal markets sell live domestic animals and/or wildlife, whereas wildlife or bushmeat markets sell non-domesticated wild animals which were either captive-bred or hunted. Live-animal and wildlife markets also sell animals for use as pets. Wet markets, in turn, sell consumption-oriented and perishable goods in a non-supermarket setting [23].

Institutional responses to the COVID-19 pandemic in the form of pandemic preparedness and response are urged to follow a deep prevention approach, through upstream prevention rather than focusing on local, national, and international spread after spillover occurs (i.e. downstream prevention) [24, 25]. However, global, national and local-level institutional responses to zoonotic threats posed by wildlife trade are diverse, variously incorporating regulatory approaches such as trade standards, regulations, and bans [15, 26]. Understanding current regulatory frameworks governing wildlife trade is an important step for effective zoonotic spillover prevention measures. Our scoping review focused on identifying and describing peer-reviewed research on regulatory approaches governing wild animal markets, including their wildlife supply chains, to clarify the role of national and global-level institutions in the prevention of zoonotic spillover and infection transmission between humans.

## Methods

Scoping reviews are an increasingly popular method to map and synthesize research evidence when the topic is of a complex or heterogeneous nature, such as the various regulatory frameworks governing wildlife trade, and when there is a need to examine the range of research activity to inform next steps of the research process. Scoping reviews share a similar structured process as systematic reviews but differ in purpose and aim. As Pham et al, and Munn et al. clarify, scoping reviews aim to provide a descriptive overview of literature in a focused area, in contrast to systematic reviews whose aim is to provide a synthesis of empirical evidence from studies assessed for risk of bias. Scoping reviews generally include a greater range of study designs and methodologies than systematic reviews focused on addressing the effectiveness of interventions (i.e. randomized controlled trials) [27, 28].

Rather than critically appraising these studies and their content, this scoping review methodology supports our research questions that aim to identify and describe existing scientific studies dealing with regulatory frameworks. Among multiple purposes for the conducting of scoping reviews suggested by Munn et al., the identification of key characteristics related to key concepts, and the identification of gaps in knowledge and policy guide the author’s choice for the scoping review methodology given the lack of standardized terminology used in policy and practice, evolving regulatory responses to the trade in wildlife, and the use of this scoping review to inform interviews conducted with practitioners as part of the larger research project [28].

This review was conducted based on the Arksey and O’Malley [29] five-stage scoping review protocol that involves: i) identifying the research question, ii) identifying relevant studies, iii) study selection, iv) charting the data, and v) collating, summarizing and reporting results.

### Identifying the research question

Panel: definition of key concepts

**Regulatory approaches** is used to describe different trade standards, regulations and regimes governing wildlife trade.

**Wildlife** are any living things that are neither human nor domesticated, including animals, fungi and plants. Wildlife includes feral animals (domestic animals living without human supervision or control), captive wild animals (with a phenotype unchanged by human intervention and living under human supervision or control) or wild animals (with a phenotype unchanged by human intervention and living without requiring human supervision or control) [30, 31].

**Wild animal markets** represent the different types of markets where wildlife trade occurs, such as live-animal markets, wildlife markets, wet markets.

**Wildlife markets** are marketplaces in which farmed or wild animals, or parts and products derived from them, are traded and sold for purposes including food, medicine, and the exotic pet trade among other purposes [32].

**Wet markets** are marketplaces that sell any fresh meat, where live animals may or may not be present, and that vary in their spatial and commercial setting, diversity of products, and customers [33].

**Zoonotic spillover** is understood as *the processes that enable a pathogen from a vertebrate animal to establish infection in a human* [34]

**Bushmeat** includes any non-domesticated terrestrial mammal, bird, reptile or amphibian harvested for food, and can include all steps in the supply chain, including the acquisition, trade and consumption of wild meat [35].

**Exotic pet**, sometimes referred to exotic animal or wild pet, is an animal of a non-native or not regularly domesticated species in the country, that is kept or traded for entertainment or company [36].

**Traditional medicine** is the sum total of the knowledge, skill, and practices based on the theories, beliefs, and experiences indigenous to different cultures, whether explicable or not, used in the maintenance of health as well as in the prevention, diagnosis, improvement or treatment of physical and mental illness [37].

This scoping review aims to identify and describe studies focused on regulatory approaches that govern domestic and international wildlife trade, to assess and clarify the role of national and global-level institutions in the prevention of zoonotic spillover and infection transmission between humans. The scoping review was guided by the following research questions:

1. What are the different regulatory approaches governing bushmeat markets, national or domestic wild animal markets for human consumption, traditional medicine, and exotic pets described in the published literature?
2. What are the different regulatory approaches governing importation of wild animals across international borders described in the published literature?
3. What are the roles of national and global-level institutions in the prevention of local zoonotic spillover and infection transmission described in the published literature on regulatory approaches governing wild animal markets?

### Identifying relevant studies

Literature searches to identify potentially relevant documents were carried out in OVID-Global Health, OVID-PUBMED, Web of Science, Scopus, and OVID-Embase electronic databases. To address current approaches, the first search of these electronic databases covered publications from January 01, 2000 until June 08, 2021. A second search of these electronic databases included articles published from June 08, 2021 until August 06, 2021. A third search of these electronic databases included articles published between August 06, 2021 until October 17, 2022. A fourth search of these electronic databases included articles published from October 17, 2022 to October 30, 2023. A search of these electronic databases was conducted on March 17, 2023 to ensure variations of the word “ban” were captured. As well, a search of these electronic databases was conducted on July 15, 2024 for additional keywords of “conservation”, “bushmeat trade”, and “bushmeat markets”.

Grey literature searches were conducted based on grey literature documents recommended by academics and practitioners interviewed as part of the larger research project. Grey literature was included because regulatory frameworks and outputs from regulatory-relevant institutions are less likely to be reported in the peer-reviewed literature compared to the grey literature and has become a common practice recommended in evidence synthesis and literature reviews [38].

The search strategy included the concepts of ‘regulatory approaches,’ ‘wild animal markets, traditional medicine, and exotic pets,’ ‘importation,’ ‘international borders,’ ‘national and global institutions,’ ‘prevention,’ and ‘zoonotic spillover.’

The full electronic search strategy and the research questions that guided the analysis of the identified articles is presented on S1 Table. A numbered table of all studies identified in the literature search, including those that were excluded from the analyses, is presented on S3 Table. A table including the name of data extractors and date of data extraction, as well as a confirmation that the study was eligible to be included in the review, is presented on S4 Table.

### Selection of studies to be included

The study followed PRISMA scoping review guidelines to systematically identify and analyze relevant studies [39].

Eligibility criteria included articles: 1) written in English, 2) published from 2000 to 2023, and 3) published as scholarly peer-reviewed literature or as grey literature. To be included, articles must discuss at least one of three points:

a) Regulatory approaches governing national or domestic wild animal trade, with uses including traditional medicine, wet markets, live markets, food, or exotic pets;

b) Regulatory approaches governing the importation of wild animals and the international wildlife supply chain;

c) The role of local, national, and global-level institutions in regulating wild animal trade for food, traditional medicine or exotic pets.

### Charting, extracting, analyzing and presenting the data

Data charting followed the process described by Pham et al. [27] The data chart was iteratively developed and reviewed by RG, RA and MW, with inputs by EG to confirm relevance and to ensure quality of the data extracted. Findings were reviewed by other researchers who discussed questions, concerns, and themes from the data charting and analysis process until consensus was reached.

Variables used for data charting and analysis were deductively derived and adapted from Vrbova et al’s (2010) systematic review of surveillance systems for emerging zoonoses [40]. The extracted data were summarized into a table using the following variables: author(s); title/year of publication; type of publication; where the study was generated; study objectives; study design and/or methodological approach; key results from study; conclusions (S2 Table). We analyzed the included articles through qualitative thematic analysis, inductively grouping studies conducted on regulatory frameworks with similar characteristics, based on the three research questions informed by the objectives of the study. Sub-themes were subsequently identified as our analysis addressed research questions, informing the identification of research gaps and recommendations for future research.

## Results

### Study inclusion

The first and second database searches yielded 620 articles. Two articles were manually added to the list of relevant studies by one author based on backward citation search. Of these, 317 were excluded due to duplication. From the remaining 303 articles, 265 were excluded after screening their relevance based on title and abstract review. Thirty-eight articles from the first and second database search and an additional 2 articles that were manually included were retrieved for full text review for a total of 40 articles. The third database search yielded 185 articles. Of these,113 were excluded due to duplication. Of the remaining 72 articles, 61 were excluded after screening their relevance based on title and abstract review. 11 articles from the third database search were retrieved for full text review. A fourth database search yielded 155 articles. Of these, 102 were excluded due to duplication. Of the remaining 53 articles, 50 were excluded after screening their relevance based on title and abstract review. 3 articles from the fourth database search were retrieved for full text review. Further electronic database searches for variations of the word “ban”, and later “conservation”, “bushmeat trade”, and “bushmeat markets” resulted in the inclusion of a further 12 articles for full text review. Grey literature searches conducted resulted in the inclusion of 22 additional grey literature documents for full text review.

**Fig 1:**
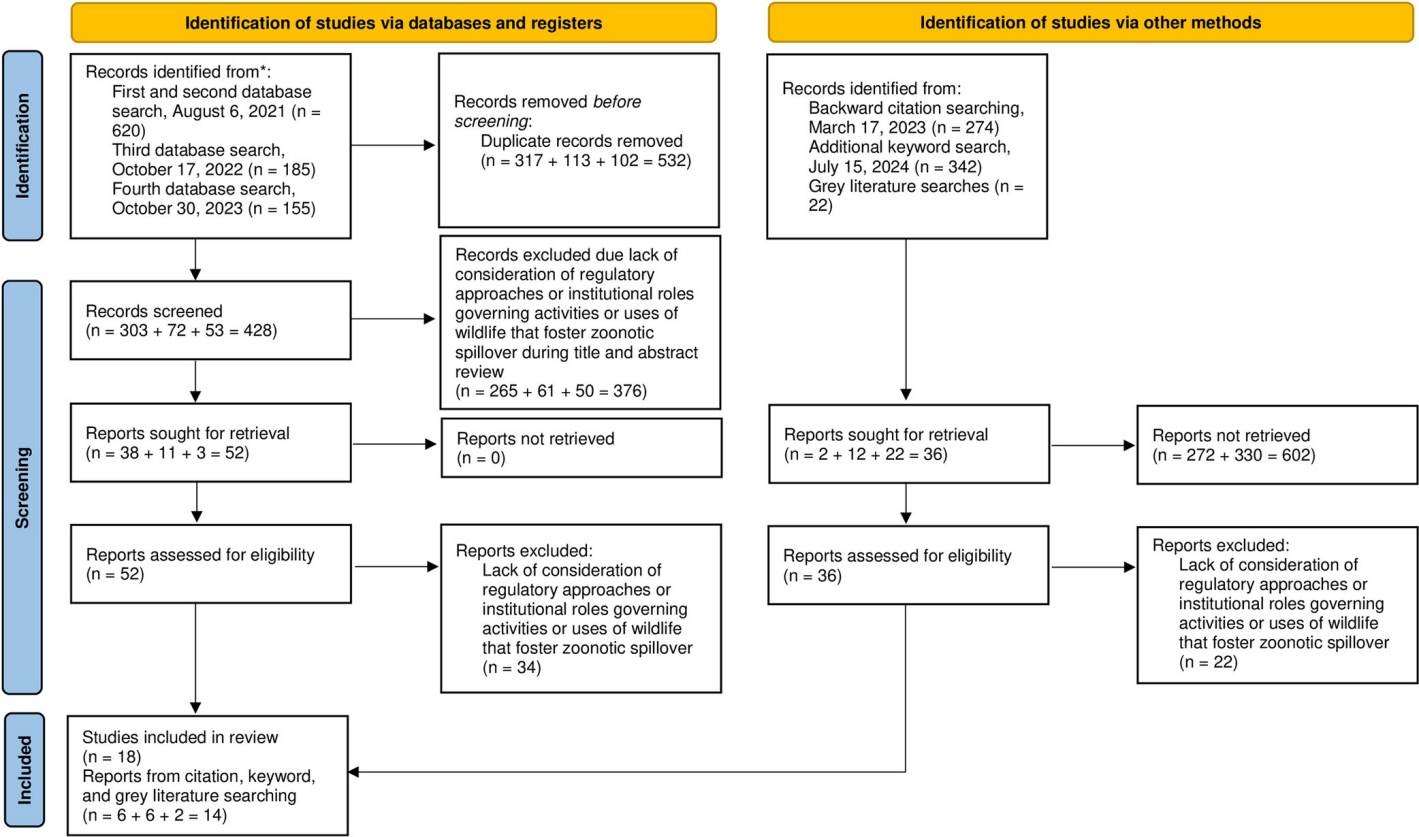
Prisma diagram.

Discussions between reviewers to further exclude articles occurred after the scoping review protocol charting table was completed for a finer understanding of the relevancy of the articles. Among the 88 articles selected for full screening, 32 articles were included in the data extraction table. A PRISMA diagram (Fig 1) illustrates how sources of evidence were selected.

### General characteristics of the studies

The results below were organized according to the research questions. Under each section, we first present relevant regulatory frameworks, before discussing how these frameworks impact the governance of wildlife trade and what challenges they face due to lack of central authority and fragmentation of policy responses.

### Regulatory approaches governing national or domestic wild animal markets for human consumption, traditional medicine, and exotic pets

#### Regulatory approaches governing wildlife trade

Regulatory approaches governing wildlife trade aim to strike a balance between supporting legitimate and sustainable trade for conservation purposes while preventing illegal trafficking and protecting threatened species. These approaches can differ between countries, but there are some common elements and international agreements that influence wildlife trade regulations, in particular the Convention on International Trade in Endangered Species of Wild Fauna and Flora (CITES). CITES is an international treaty that came into effect in 1975 and is one of the most crucial regulatory frameworks for wildlife trade. Its goal is to ensure that international trade in wild animal and plant species does not threaten their survival. However, individual countries enact their own wildlife laws and regulations to govern domestic trade and protect their native species. These laws may specify which species are protected, which are allowed for trade, and the permits or licenses required for legal trade.

Establishing the legality of wildlife trade can be difficult, however. For Toland et al. [41], the distinction between legal and illegal trade between countries can be problematic, as trade considered legal or illegal in one country may be the opposite from the viewpoint of another country’s legislation. Romero-Vidal et al. [42] found that the legal export of parrots may be maintained while the domestic capture of parrots as companion pets is prohibited among neotropical countries (approx. 46 countries in Central and South America, including Mexico); or parrots as crop pests may be legally hunted while keeping parrots as pets is prohibited. In some cases, issues are also in evidence domestically: wildlife trade regulations in China were found not to function as expected given outdated protected species lists, insufficient cross-sector collaboration, and weak restrictions and law enforcement for the farming and trading of species [43].

China’s 2020 decision to ban the trade and consumption of terrestrial wild animals [19] also features inconsistencies between China’s Protected Species List and the extinction risks set by CITES and the International Union for Conservation of Nature (IUCN) [44], demonstrating the gap between the scientific justifiability of the ban on conservation grounds [45]. This ban excluded species with zoonotic spillover potential such as bats, which raises concerns as bats are the suspected reservoir of SARS-CoV-2 and the natural reservoir for a range of coronaviruses [44], and highlights the need for research addressing the nexus between bushmeat, wet markets and disease within a One Health (OH) framework [46].

Wildlife trade also underlies traditional medicine and exotic pets. The United Nations Environment Program (UNEP) and International Livestock Research Institute (ILRI) cite global trade in traditional medicine and exotic pets as one of the reasons for the expanding human-animal-environment interface within which zoonotic spillover is facilitated [20]. Toland et al. and Neuwirth et al. [41, 47] argue that the regulation of traditional medicines and exotic pets is plagued by problems of fragmentation and a lack of oversight.

Neuwirth and Svetlicini [47] highlight the patchwork nature of international law (eg, conflicts of laws) and compare this to the subdivision and specialization of international legal regimes. Viens et al. [48] similarly find a lack of consensus and devolution of the definition, categorization, and governance of animals within international law to different individual governance systems, leading to a fragmented approach to the regulation of human-animal interactions related to zoonoses.

One of the biggest challenges for governing wildlife trade is the fragmentation of authority and ambiguity of regulation. For Huang et al. [44], China’s 2020 Wildlife Protection Law provides ambiguous legal provisions where restricted wildlife may still be used commercially for fur, traditional medicine, and captive breeding. Similarly, Chen et al. [49] find that a lack of specificity in defining what is exempt from China’s ban on trade in ivory may enable the legal importation and trade in ivory despite such items being illegal. D’Cruze and Macdonald (2015) call for extending the ban on the Asian big cat trade to include commercial trade in captive bred individuals given the increase in illegal poaching of clouded leopard parts for traditional medicine [50]. To address the fragmentation and lack of oversight in the global trade of amphibians as pets, Borzee et al. [26] recommend regulating the movement of such amphibian species that can spread pathogens through the global pet trade to prevent zoonotic spillover.

#### Regulatory approaches governing human consumption of wildlife through bushmeat trade

The regulation of domestic bushmeat hunting, farming, selling, trading, purchasing, and use varies in legislative environment and legal requirements according to each country. Despite the existence of legislation to prohibit the importation of bushmeat across countries, regulatory efforts are hindered by gaps in enforcement and a lack of consensus on terminology.

Chaber et al. [51] notes that animal health legislation and wildlife trade legislation exists in the European Union that prohibits the import of bushmeat from third-party countries into the EU where such bushmeat belongs to CITES annex-listed species. However, the authors note that there is no general established practice in the EU for seizing illegal bushmeat, making the enforcement of these regulations through investigation of alleged offenses and securing evidence for prosecution difficult.

Svensson et al. [52] found that there is some level of protection for galago primates, a species that is consumed as bushmeat, in the respective national legislations of countries in the galago range as per the countries’ CITES obligations. However, authors found that galago trade regulations vary in protection due terminology issues resulting in significant differences in meaning, outdated and/or incorrect taxonomy within respective legislation, and due to the reliance on subsidiary regulations that have not been implemented and/or administrative agencies that were not yet formed [52].

In Colombia, Van Vliet et al. [53] found that commercial bushmeat hunting continues despite the de facto inability to obtain commercial permits for bushmeat hunting because of conflicting legal authorities, a lack of technical capacity, and a lack of guidance. Autonomous Regional Corporations are unable to exercise their legal authority to refuse the issuing of commercial hunting licenses in their jurisdiction because the national Ministry of Environment has not published a list of species permitted to be harvested within a given global harvesting quota, of which the Ministry of Environment is entitled to set by national Decrees. The national Ministry of Environment and the Autonomous Regional Corporations have also not developed the formal criteria and methodologies to monitor hunted species and to estimate the available stocks that are required to issue hunting licenses. Furthermore, sanitary regulations for the handling of bushmeat and its processing are yet to be defined by the Ministry of Health and Social Protection, such that bushmeat cannot be legally commercialized for human consumption [53].

In the city of Sekondi-Takoradi, Ghana, bushmeat trade was found to be largely unregulated by state or local institutions, with no evidence of any individual actors or actor groups exerting control over the bushmeat market [54]. Absent actors with regulatory functions include the local police which issue licenses for shotgun use, the wildlife department which issues licenses for bushmeat hunting, district assemblies which issue licenses for bushmeat trade, third-party actors such as hunter unions, guilds, or associations that would provide some level of regulation, representation, and arbitration [54].

In Southern Africa, various transfrontier conservation area structures exist to promote cooperation amongst multiple countries in the management of contested shared natural resources, including wild animals, migratory species, and pastures for livestock rearing [55].

The existence of such transfrontier conservation area structures demonstrates functional collaboration among stakeholders [55], however transfrontier conservation areas in sub-Saharan Africa may increase the risk for incursion of Trichinella spp. into the human food chain by increasing the association of wildlife with domestic animals in rural areas at the edge of protected areas, and therefore the risk of Trichinella spp. infection in humans [56].

#### Regulatory approaches governing use of wildlife for traditional medicine trade

The following section relies on the single study found on regulatory approaches governing use of wildlife for traditional medicine trade. Table 1 below summarizes information describing the international legal regimes regulating traditional medicine.

**Table 1:** The international legal regimes that regulate traditional medicine.

Type of law	Source of governance/regulation	Purview	Degree of legal harmonization^1^
1. International trade law	The World Trade Organization (WTO) framework	Rules on goods and services	High
2. International intellectual property rules	WTO Agreement on Trade-Related Aspects of Intellectual Property Rights, and the World Intellectual Property Organization Conventions	Copyright, trademarks, and patents, intangible cultural heritage or traditional knowledge	High
3. Competition or anti-trust law	National and regional competition rules	Regulate unfair trade practices and methods of competition	Low
4. International health law	Governed by the World Health Organization (WHO)	Traditional and conventional medicine	Low
5. International private law	National rules on conflicts of laws	Governing the international activities between private persons	Low

^1^ Legal harmonization refers to how medical and health goods are regulated (e.g. unified or fragmented regulation/legislation) and the level (e.g. national, regional, global) [47].

Regulatory approaches governing use of wildlife for traditional medicine trade aim to address the potential impacts on wildlife conservation and animal welfare while respecting cultural practices and traditional knowledge. The trade of animal parts and derivatives for use in traditional medicine has been a significant concern due to its contribution to illegal wildlife trafficking and threats to endangered species.

Traditional medicine is increasingly dominated by western governance arrangements that regulate relations between health, trade, and intellectual property rights, and, as such, is subject to similar degrees of harmonization and fragmentation [47]. CITES also plays a crucial role in regulating the international trade of animals and plants used in traditional medicine. The treaty categorizes species into different classes (Appendices I to III), with varying levels of protection, and stipulates that permits are required for the international trade of species listed under Appendix I and II, ensuring that trade is legal and sustainable. In addition, some countries maintain positive lists that identify which animal parts and products can be legally used in traditional medicine. These lists often exclude endangered species and only include species that are abundant and can be sustainably sourced.

Traditional medicine has been described by Neuwirth and Svetlicini [47] as regulated within a public health environment where the dominant Western pharmaceutical model of health care increasingly includes the practice of traditional medicine, now categorized as “medicinal products”. The authors pose that the globalized nature of the market for medicinal products and other health-related goods converge as two distinct models of health care (i.e. a Western pharmaceutical model of health care and traditional medicine). Finally, the authors do not link traditional medicine or traditional medicines containing or being derived from animals or animal parts with zoonotic spillover, zoonotic infection transmission.

#### Regulatory approaches governing exotic pet trade

Legislation concerning both the trade and possession of exotic pets in the European Union, the United States, and Canada is analyzed by Toland et al. [41] who highlight the fragmentation and lack of oversight by a single authority. Authors linked trade in exotic pets and their ownership with zoonotic infection transmission, but not zoonotic spillover (see panel for definition), where they define zoonotic infection transmission as the incidence or end result of human infection with animal-based diseases (e.g. salmonellosis) [41].

The CITES is also the primary instrument regulating the international trade in exotic pets [41] included in their list. The Convention identifies wildlife species that may be legally traded, in what quantities, and under which conditions (idem), whereas the Bern Convention on the Conservation of European Wildlife and Natural Habitats and the Bonn Convention on the Conservation of Migratory Species of Wild Animals strengthens the legally binding nature of provisions within CITES for states that are a party to both conventions.

At the national level, legislation regulates trade in exotic pets through implementation of the above conventions and, where applicable, further limits the human-exotic pet interface from hunting to possession or ownership [41]. Such additions to international standards are permitted so long as the national standards are more stringent and scientifically justifiable [45].

Although national-level regulations are therefore compatible with the minimum required by international regulations, Toland et al. [41] argue that a lack of harmonization exists regarding which species’ trade is expressly prohibited, resulting in a legal gap where some species’ trade are targeted systematically, and prohibited only nationally and not internationally by CITES. This can be observed in the case of continued international trade in the Australian shingleback lizard [57] despite strict regulation of the species’ trade domestically in Australia, and no international recognition of this domestic protection.

There is also a lack of inter-state harmonization for regulations regarding the trade in exotic vertebrate pets between the domestic and national levels. Pratt et al. [58] found that state-level governments in the United States have implemented regulations that permit trade in exotic vertebrate pets that are banned from international/national import into the United States due to public health and conservation concerns. Once exotic vertebrate pets have been imported into the United States, inconsistent domestic regulations facilitate the movement of exotic vertebrate pets that pose substantial invasion and disease risks (ibid). Pratt et al. [58] also found that definitions and classifications for regulating different vertebrate taxa varied greatly across state-level governments in the United States, and the terms ‘pet’ and ‘companion animal’ were poorly defined and inconsistent across states, further facilitating the continued possession of exotic pets in states where these animals are banned.

In the case of the poaching and international trade in parrots, differences in the timing of national implementation of international wildlife legislation (i.e. CITES) varied between countries in Central America, the Caribbean, and South America, allowing protected parrot specie trade, keeping as pets, and poaching, presumably until countries ratify and implement the Convention [42].

Beyond the potential incompatibility between national and international regulations, challenges in the regulation of the exotic pet trade are similar to those identified above for wildlife and traditional medicine trade, in particular the fragmented nature of the regulatory landscape.

### Regulatory approaches governing importation of wild animals

Table 2 summarizes institutions, conventions and environments that govern importation of wild and captive bred wild animals across international borders.

**Table 2:** Institutions, conventions and environments that govern importation of wild and captive bred wild animals across international borders.

Institution/ Conventions	Mandate	Instrument	Impact	Effect
World Trade Organization (WTO)	Ensure free trade	Legally-binding	Sets regulations and standards related to trade generally. Provides dispute settlement for international trade.	Harmonize national regulations and standards. Provide incentives related to free trade.
Convention on the International Trade in Endangered Species of Fauna and Flora (CITES)	Wildlife species survival	Legally-binding (as per WTO Sanitary and Phytosanitary Agreement)	Set quotas for wildlife trade. CITES Appendices Significant trade review and other recommendations.	Harmonize national regulations and standards.
World Organization for Animal Health (WOAH)	Animal health and welfare	Legally-binding (as per WTO Sanitary and Phytosanitary Agreement)	Set standards for animal health and welfare. Set standards for diagnostic tests and vaccines. Evaluate and analyze national veterinary services.	Harmonize national regulations and standards.
International Union for Conservation of Nature (IUCN)	Animal movement (internationally) and animal welfare	Non-legally-binding	Provides guidelines and best practices.	Influence national regulations and standards
International Plant Protection Convention (IPPC)	Limit invasive alien species	Legally-binding (as per WTO Sanitary and Phytosanitary Agreement)	Provides standards and risk analysis guidance.	Harmonize national regulations and standards
Convention on Biological Diversity (CBD)	Limit invasive alien species; protect biodiversity	Legally-binding, yet lacks compliance measures	Provides guidelines and best practices.	Harmonize national regulations and standards.

The WTO has the overarching responsibility for regulating international trade, including trade in wildlife [45]. Through the WTO’s Sanitary and Phytosanitary (SPS) Agreement, certain international organizations are recognized as the reference organization regarding standard-setting related to trade. However, the WTO, through the SPS Agreement, does not ensure state compliance with the reference institution’s standards, but is concerned with preventing unnecessary disruptions to trade. The SPS Agreement prevents national measures that derogate or exceed those measures of the reference body [45]. That is, the reference organizations maintained in WTO and newer bilateral or regional trade agreements only have legal authority once states’ measures exceed those of the reference organizations and cannot meet the various science-based thresholds. Where wildlife-related agreements and regulatory standards (e.g. CITEs, Codex Alimentarius—CODEX, World Organization for Animal Health—WOAH, International Plant Protection Convention–IPPC, Convention on Biological Diversity—CBD) are discussed in new bilateral or regional trade agreements, non-compliance by parties to these trade agreements are not subject to dispute resolution, and are thus unenforceable [59]. Therefore, the extent to which reference organizations regulate the importation of wild animals across international borders is largely realized by the WTO through standard-setting based on the SPS agreement [45].

Similar to the governance of traditional medicine and exotic pets, oversight for importation of wild animals is spread throughout a few organizations. Based on the findings of Cooper and Rosser [45], two broad groups of institutional mandates exist for regulating the importation of wild and captive bred wild animals each with their own regulatory environment: wildlife survival and biodiversity protection.

#### Institutional mandates: Wildlife species survival, animal health and movement

CITES regulates international wildlife trade to support wildlife species**’** survival. CITES categorizes wildlife species into three appendices depending on the risk that international trade would have on the species’ survival. Based on these appendices, the tradable quantities (i.e. quotas) of wildlife species and their products are determined. Each of these CITES appendices has associated permits and other requirements which must be met for international trade to be legal and non-detrimental to the species’ survival [60]. The appendices, their descriptions, and relevant importation requirements can be found in Table 3. CITES also conducts a significant trade review, which reviews Appendix II (listed species traded in significant quantities), and provides recommendations for the species listed in Appendix I [60]. In keeping with the SPS agreement, states are obligated to base their relevant national measures on the standards developed by CITES.

**Table 3:** CITES appendices [45].

Appendix	Description	Importation Requirement
I	Includes ‘all species threatened with extinction which are or may be affected by trade’.	• Import and export permit or re-export certificate. • If introduction from the sea, certificate. • Non-detrimental finding required.
II	Includes ‘all species which although not necessarily now threatened with extinction may become so unless trade in specimens of such species is subject to strict regulation’.	• Export permit or re-export certificate. Many countries also require an import permit. • If introduction from the sea, certificate. • Non-detrimental finding required.
III	Includes species listed [unilaterally] by parties as being subject to regulation within their jurisdiction and for which international co-operation is needed to control trade.	• Certificate of origin or export certificate. • Non-detrimental finding not required.

By defining enforcement as the ability to ensure compliance, Cooper and Rosser [45] determined CITES’ enforcement ability as successful due to trade sanctions that moved parties towards compliance in writing. A caveat is that compliance requirements are low, and many parties only meet CITES’ basic requirements. In the case of seahorses, for example, Foster and Vincent [61] found that the CITES’ significant trade reviews lack specificity and metrics for tracking compliance with Review recommendations, and are based on the precedents set by other species.

Developing guidance that ensures consistency in the implementation of CITES provisions as well as expanding legal definitions and approaches would address such compliance issues [26, 62–64]. Foster and Vincent [61] recommend 1) the focus of the CITES Review be shifted from outputs to outcomes and impacts; 2) match CITES Review recommendations to the needs and capacities of the Parties; 3) give experts a formalized role in the CITES Review process; and 4) create funding mechanisms for the Review process, the integration of experts in the Review process, and for assisting Parties in addressing the recommendations from the Review. However, Thiermann [65] argues that global commitment and political will are needed to address compliance issues rather than additional science-based international standards or improved dispute resolution mechanisms.

The WTO recognizes WOAH (formerly OIE) as the reference organization for zoonoses and terrestrial and aquatic animal health in international trade [45]. The WOAH sets animal and aquatic animal health international trade standards for wildlife products to ensure animal health and welfare. While WOAH is explicitly concerned with zoonoses, the focus is more on controlling re-emerging events and surveillance rather than preventing the spillover of new or emerging zoonotic pathogens from being transmitted [65]. The various WOAH documents, their descriptions and relevance can be found in Table 4. In keeping with the SPS agreement, states are obligated to base their relevant national measures on the standards developed by WOAH.

**Table 4:** WOAH regulations, standards, and evaluation for international wildlife trade [45, 66].

Document	Description	Relevance
International [Terrestrial] Animal Health Code and Manual	Outlines standards regarding the prevention of specific animal disease transmission, including laboratory operation, diagnostic procedures, and production sectors	Applies to mammals, birds, and bees Eg, veterinary certification
International Aquatic Animal Health Code and Manual	Outlines standards regarding the prevention of specific animal diseases transmission, including laboratory operation, diagnostic procedures, and production sectors.	Applies to fish, molluscs, and crustaceans farmed or released
Manual of Standards for Diagnostic Tests and Vaccines	Outlines the guidelines for the standardization and quality control of diagnostic tests and vaccines related to wildlife.	Applies to wildlife
Performance of Veterinary Services Evaluation and Gap Analysis Tools	Evaluate, analyze, and enhance the national performance of veterinary services to control animal diseases and zoonoses.	Application and submission to the tool is voluntary, i.e. non-legally binding.

Finally, IUCN (formerly World Conservation Union) regulates the international wildlife trade through the provision of guidelines and best practices related to the movement and welfare of animals [45]. However, the IUCN’s guidelines are non-legally binding, and thus, the implementation of and compliance with such standards are less likely than with instruments that provide economic or reputational repercussions for non-compliance. Cooper and Rosser [45] list the following documents as the relevant guidelines and best practices of the IUCN:

1. IUCN Guidelines for the Prevention of Biodiversity loss caused by Alien Invasive Species
2. IUCN/Species Survival Commission Guidelines for Re-Introductions and Other Conservation Translocations
3. IUCN Guidelines for the Placement of Confiscated Animals Closely Related to CITES Resolution Conf. 10.7: Disposal of Confiscated Live Specimens of Species
4. IUCN Policy Statement on State gift of animals
5. IUCN Position Statement on Translocations of Living Organisms: Introductions, Re-Introductions, and Re-Stocking
6. IUCN Policy Statement on Captive Breeding

#### Institutional mandate: Limit harmful introduction of invasive alien species

While plant pathogens have been widely overlooked in discussions about global disease spread, Kim et al. highlight instances of illness in humans caused by various bacterial phytopathogens, and the potential for opportunistic pathogens to develop infection strategies of spillover across kingdoms, challenging the notion that plant pathogens are not causative agents of disease in humans and animals [67]. This led Kim et al. to consider the trade in plants and to include the IPPC in their analysis.

The IPPC regulates the international wildlife trade by developing standards to prevent the introduction, spread, and establishment of plants that could become invasive [68]. Such standards may be found in the *International Standards for Phytosanitary Measures*, which also include glossaries and risk analysis guidance [68]. While the Convention is only legally binding for those states who choose to be a party to the Convention, WTO parties must base their national measures on the standards developed within the Convention as per the WTO SPS Agreement [45]. The IPPC is recognized by the WTO as the reference organization for phytosanitary standard-setting related to trade [68].

With regard to the prevention of zoonotic spillover to infection transmission between humans, Zhang et al. and Chinchio et al. [69, 70] highlight potential for zoonoses from introduced alien species. In order to effectively prevent invasion risks and indirectly prevent zoonotic spillover, Reino et al. argue that global bans are needed as opposed to current regional bans such as those in the European Union [71]. Towards limiting the harmful introduction of invasive alien species, the IPPC cooperates with the CBD. The CBD encourages state parties to regulate the international movement of genetic material to both limit invasive alien species (IAS) and to protect biodiversity, thereby affecting the regulation of the international wildlife trade [45]. While legally binding, the Convention has taken a ‘soft’ approach toward implementation, developing instruments that are not backed by legal obligations to implement [72]. Both the IPPC and the CBD do not directly address the prevention of zoonotic disease, although their respective mandates are part of the trade standards, regulations and incentives that govern the international wildlife trade.

### Role of national and global regulatory institutions in the prevention of zoonotic infection

The findings below emerged from the broader groups of institutional mandates found in the previous section. We assess the roles of institutions in preventing zoonotic spillover transmission by considering how their mandates are being fulfilled. By setting international standards to guide national practice, these institutions largely inform local governance concerning the prevention of zoonotic infection.

Overall, the findings indicate that a mandate to prevent zoonotic spillover was not always explicitly the focus of institutions and their respective regulatory systems. While such regulation would contribute to zoonotic spillover prevention, it would do so indirectly and through other pathways (e.g. by promoting animal health).

#### Indirect prevention of zoonotic spillover; prevention of infection transmission between humans (WOAH, FAO, WHO)

The United Nations Food and Agriculture Organization (FAO), WHO and WOAH engage directly in the prevention of zoonotic infection transmission through tools aimed at surveillance, prevention and containment. The Global Early Warning System for Animal Disease is a surveillance system that tracks animal diseases and zoonoses as a joint WOAH/FAO/WHO initiative [73]. Through the International Health Regulations (IHR), the WHO provides a legally binding instrument for state parties to notify, assess, and contain public health emergencies of international concern (PHEIC) [64]. While the IHR do not expressly mention zoonotic disease, the scope of what constitutes a PHEIC is broad so as to cover any incident involving the international spread of diseases requiring a coordinated international response, including zoonotic spillover and resulting infections [64].

#### Regulation of the movement (trade) of wildlife (CITES, CBD)

Institutions may prevent zoonotic infection transmission through their role in regulating the international movement of flora and fauna. CITES regulates the international wildlife trade through its three appendices (see Table 2) that separate wildlife species according to requirements for import, export, and re-export to address species survival in international trade [62]. Although CITES has no mandate over zoonoses, by regulating the trade and movement of wildlife, it influences factors that may lead to zoonotic spillover, such as animal welfare and biological diversity [34]. The CBD regulates the international movement of genetic material to protect the biological diversity of countries through the similarly named Convention [45]. In parallel, the IPPC controls and prevents the international spread of pests (invasive alien species) and plant diseases [68].

#### Harmonization of state practice with international standards, policy and practice (WOAH, CITES, WTO, WHO, IUCN, IPCC)

Institutions prevent zoonotic infection transmission through their role in setting international standards, which guides legislation and practice of state parties and non-governmental organizations. WOAH sets international trade standards (trade quotas) through the Terrestrial Animal Health Code and the Aquatic Animal Health Code [45]. Similarly, CITES provides legally-binding requirements for the international trade of wildlife within the CITES appendices, as mentioned earlier. State parties are legally obligated to consult and base their practices and legislation on the respective WOAH and CITES guidelines, depending on the commodity traded and the animal disease(s) of concern [45]. IUCN provides guidelines on aspects related to the movement and welfare of wildlife within international wildlife trade [45]; these guidelines are however non-legally binding and non-enforceable.

In March 2024, CITES and WOAH signed a Memorandum of Understanding [74], catalyzing their collaboration to reduce the risk of zoonotic disease emergence associated with international wildlife trade through a joint programme of work focused on training, capacity-building, networking, coordination and communication. Key areas of mutual interest include the safe, traceable, and legal international trade in wild species of animals, identification of zoonotic disease risks associated with CITES listed species and activities, and facilitation of secure transport for biological samples from wild animals. In May 2024, WOAH published voluntary guidelines for addressing zoonotic spillover risk in wildlife trade spanning the sub-national, national, regional, and international scales [30]; wildlife, domesticated animals, and humans spanning the entire supply chain in a wildlife trade system including wild animal markets, wildlife markets, wet markets; and uses including bushmeat, exotic pets, and traditional medicine. A framework is provided for authorities with a mandate related to animal health and welfare, public health, or wildlife management and trade to assess risk and identify risk-management strategies for wildlife trade guided by a precautionary approach.

The IPPC sets standards related to the environment and biodiversity to help state parties harmonize their management of invasive alien species related to plants [68]. In this way, the role of the IPPC transitions from the standard setting role of institutions such as the WOAH and the CITES Secretariat to the harmonizing and coordinating roles of the WTO and the Codex Alimentarius Commission.

The WTO enforces the harmonization of national measures with international animal health laws and trade rules by providing dispute settlement for trade disputes between state parties [45]. The Codex Alimentarius Commission promotes the coordination of the food standards undertaken by international governmental and non-governmental organizations [68]. The WHO’s IHR may be viewed as international standards regarding disease surveillance, notification and control that direct state action given their legally binding nature [64].

#### Information sharing and disease control capacity building (WOAH, CITES)

Institutions prevent zoonotic spillover and infection transmission among humans through their role in facilitating capacity building for states, especially concerning disease prevention surveillance and control efforts. WOAH facilitates capacity building for states, especially in mainstreaming veterinary services and veterinary public health as a key part of the national and international response to the international wildlife trade and animal disease prevention and control, as well as information sharing amongst state parties regarding expertise and best practices [73]. Similarly, WOAH certifies the competence and credibility of national veterinary services through the Tool for Evaluation of Performance of Veterinary Services (WOAH-PVS Tool) and facilitates capacity building in national veterinary services to promote trust amongst international trading partners and ensure the international wildlife trade fosters public and animal health [73]. TRAFFIC, the Wildlife Trade Monitoring Network, acts as a source and facilitator of research and data regarding the trade status of, and threats to CITES species; reports on prosecutions for illegal trade; and CITES enforcement activities globally [45].

## Discussion

This scoping review aimed to identify and describe studies dealing with regulatory approaches governing wildlife trade to assess the role of national and global level institutions in the prevention of zoonotic spillover and infection transmission between humans. Findings addressing the three research questions are discussed below.

### Regulatory environment governing national wild animal markets for human consumption, traditional medicine and exotic pets

Our review of regulations governing wildlife trade found evidence of temporal mismatch between countries adopting CITES protected species lists, and of partial mismatch between the CITES protected species list and domestic lists [19, 43, 44]. Future research on countries facing similar issues could assess the effectiveness of precautionary interim risk management approaches that adopt updated CITES lists, while domestic lists are updated and legislation is ratified and implemented.

Within the environment governing traditional medicine, the WTO is the main regulatory organization, providing the basis for two of the identified five regimes: international trade law and international intellectual property rules. These trade law and intellectual property systems also reflect a high degree of international legal harmonization, granting the WTO recognized authority on international trade as Cooper and Rosser emphasize [45]. However, a focus on the primacy of the WTO in regulating traditional medicine would neglect the role of the WHO and international health law. While the dynamics of health and trade as two distinct approaches are considered within the broader literature on health and trade [75, 76], the complex regulatory environment described in our findings–and broader intersectoral OH linkages between wildlife trade and complex socio-ecological interactions that produce the vulnerable interfaces where zoonotic spillover occurs—tend to be oversimplified [77].

The same applies to the dynamics of conservation and the risks posed by animal trade for traditional medicine purposes, especially given the renewed interest in regulating wildlife trade given the SARS-CoV-2 pandemic, which likely reflects a similar zoonotic spillover process that led to the SARS pandemic [15, 78]. However, trade and health and trade and conservation, as competing interests, demonstrate the patchwork nature of regulation in traditional medicines and exotic pets.

Organizations and regulations emerging to address regulatory gaps designed to strengthen extant regulations and standards face challenges harmonizing arrangements that reflect different institutional mandates and jurisdictional priorities. This is evident in the exotic pet regulation with the Bern Convention on the Conservation of European Wildlife and Natural Habitats and the Bonn Convention on the Conservation of Migratory Species of Wild Animals strengthening CITES provisions, where trade of certain species might be legal at the national level, but illegal at the international level [41].

It may, however, be argued that international trade law, international intellectual property rules, and competition or anti-trust law may be combined, given these different foci relate to trade [79]. More generally, no singular supranational agency exists to coordinate amongst these identified legal regimes to regulate traditional medicine. In the case of exotic pet regulation, it may be argued that compliance and enforcement challenges are responsible for such legal pluralism [80].

While animal products are used in traditional Chinese medicine which has been increasing in recent years [81], our results support the general finding that the role of animals and animal products in traditional medicine is neglected [82] or not systematically studied [83] in the literature.

Neuwirth and Svetlicini [47] use the term Traditional Chinese Medicine to operationalize what they refer to as “traditional Oriental medicine,” thereby obscuring differences in the culture, history, knowledge, and context between different countries and their Indigenous peoples, including Malaysia, Singapore, Australia and Hong Kong. This reflects the lack of clear distinction between these various forms of medicines, perhaps promulgated by the current international regulation of traditional medicine. Neuwirth and Svetlicini [47] found traditional medicine was broad enough to include traditional Chinese medicine as a subset, in the regulation of medical products and treatments generally. The lack of sources found in the academic and grey literatures on regulatory environments governing traditional medicine trade suggests a need for further research in this area to situate our findings within global and state scales and contexts.

The partial coordination of exotic pet regulation at the national/sub-national and international levels as found by Toland et al. [41] may also suggest lack of research that considers ecosystems guided by a OH approach [84, 85]. Future research on trade in exotic animals and pets from a OH and biodiversity lens could inform future regulatory approaches governing this dimension of wildlife trade.

### Regulatory environments governing bushmeat trade, importation of wild and captive bred wild animals across international borders for food

The literature identified suggests governance gaps related to incomplete institutional purviews and a lack of express mandates for preventing zoonotic spillover. Reeve [60] notes that CITES does not address the markets where wildlife is sold, nor provides for the prevention of zoonoses. Similarly, the WOAH mandate does not expressly consider the control of invasive alien species or the protection of biodiversity, despite a broad purview [68]. While Khan and Pelgrim [68] note that WOAH contributes to these ends through an animal health perspective, the IPPC’s purview is also relatively limited given its focus on plants.

The purviews of WOAH and IPPC complement each other in a patchwork manner, as organizations emerge to address the gaps in the current regulatory environment. The effects of incomplete institutional purviews also explain issues related to enforcement of, and compliance with international standards, regulations and incentives.

The regulation of human consumption of wildlife through bushmeat trade presents similar regulatory gaps and enforcement challenges. In European Union countries with CITES-listed species, problems identified include prosecuting offenses and inconsistent practices in seizing illegal bushmeat [51]. Countries outside the European Union with existing approaches governing bushmeat hunting [53] and countries that follow CITEs regulations [52] still face problems with varying terminology and taxonomy issues, insufficient technical capacity and subsidiary regulations.

Domestic CITES-implementing laws do not cover all wildlife species but only those within the CITES appendices, and the liability for violating such national legislation is not tailored to the specifics of the violation (e.g. types of offenders and their resources, the circumstances and level of involvement of the offender), nor coordinated internationally [60]. The lack of coordination may hinder the effectiveness of national and international wildlife trade bans aiming to prevent zoonotic spillover.

Similarly, WOAH Listed Diseases include only those established as relevant to wildlife and international trade (i.e. of economic utility). That is, the reporting of non-WOAH listed diseases is voluntary, and new and emerging infectious diseases are not expressly considered. What the regulatory frameworks lack is the capacity to prevent new pathogens from emerging in the first place [24]. Further research considering the impact of incomplete or fragmented regulatory purviews on domestic bushmeat trade, wild animal and captive bred wild animal importation, and zoonotic spillover could inform enhanced governance approaches to prevent zoonotic spillover events.

### National- and global- level institutional coordination in preventing zoonotic infection

Despite a common set of institutional roles in the prevention of zoonotic spillover and transmission, organizations had differing degrees of influence and agency with respect to a given role. The FAO, WHO and WOAH engage in preventative, surveillance, and containment measures, while CITES considers import/export requirements to protect species survival. These findings are supported by Viens et al. [48] analysis of legal instruments related to handling of animals that pose a zoonotic risk. “*The international legal instruments that these governance systems use reflect different ways of viewing and treating animals*, *which has led to a similarly fragmented approach to the regulation of human–animal interactions related to zoonoses”* [48]. Information-sharing and capacity building are important aspects of zoonotic prevention efforts that could be used to address these inconsistencies. Future research could explore the extent to which information sharing mechanisms among global and national level organizations support the harmonization of wildlife trade regulations preventing zoonotic infection.

We found no evidence of formal logic in how zoonotic infection prevention roles were divided between global and national organizations. Organizations had mandates to fulfill the regulatory gaps that emerge within the broader environment (i.e. its constituent organizations and treaties), particularly after a critical juncture that would fundamentally alter society, such as the SARS-CoV-2 pandemic. Given such a patchwork design, confusion and lack of coordination would result. Future research could mobilize a OH approach to improve information sharing and intersectoral governance and policy coordination that contributes to a better understanding of the role of global and national organizations in preventing zoonotic spillover and transmission.

## Limitations

This review included published studies and grey literature in the English language only and publication bias is a possible limitation. The grey literature considered was from those international organizations referred to by academic and practitioner interviewees. We addressed this by having two reviewers (RA and RG) reach consensus on sources to be included and the analysis of thematic content.

The lack of a universal standard for terminology used in describing and classifying wildlife, and in defining what constitutes traditional medicine, wild animal markets, wildlife markets, and wet markets reflects conflicting perspectives between academics and practitioners in various policy domains (e.g. public health versus biodiversity conservation versus food security versus trade) and variability between countries and their policies and practices (Gallo-Cajiao et al., 2023). This lack of standardization in terminology presents a risk of bias in our research questions, identification of relevant studies, and findings. In particular, a possible limitation is that our search strategy did not differentiate between traditional Chinese medicine and traditional medicine. In addition, our lack of findings regarding regulation of traditional medicine to prevent zoonotic spillover may reflect a limitation of our search strategy of including only articles written in English, or it may suggest that animals and animal products used in traditional medicine are not distinguished within international regulation, nor regulated towards the prevention of zoonotic spillover.

## Conclusion

Reducing the probability of spillover events is key to prevent future pandemics. Regulatory measures aimed at reducing the human footprint in natural systems–including but not limited to wildlife trade [86, 87]—and emphasizing the inclusion and separate funding of regulations and measures that address ecology, conservation, and wildlife biology related to spillover prevention in multilateral cooperation fora [86, 88, 89]. Spillover prevention measures also involve the adoption of varying degrees of wildlife trade bans [22, 90]. Such recommendations consider the trade-offs in livelihoods and food security to protect global biodiversity and significantly reduce the risk of zoonotic spillover and transmission [21, 91].

Our review found instances where the institutional and regulatory purview of zoonotic spillover prevention in the wildlife trade is characterized by fragmentation and lack of oversight by a single coordination authority. The lack of harmonization within and between national and supranational regulations reflects competing interests and organizational mandates across jurisdictions and sectors. Effective policy responses must take the differing relative zoonotic spillover risks in wet markets, live-animal markets, and wildlife markets into account.

Enhanced coordination and collaboration for prevention of zoonotic infection and spillover and infection transmission may be informed by future research focusing on the effectiveness of timely information sharing and global- and national- level harmonization of wildlife trade regulations.

## Supporting information

S1 Table (DOCX)

S2 Table (XLSX)

S3 Table (XLSX)

S4 Table (XLSX)
