# Supplementary material for: Preventing zoonotic spillover through regulatory frameworks governing wildlife trade: A scoping review
Source: PLoS One. 2025 Jan 6;20(1):e0312012. doi: 10.1371/journal.pone.0312012 (PMC11703008; doi:10.1371/journal.pone.0312012)
Supplement: S1 Table — (DOCX) [file pone.0312012.s001.docx]

| **Search Question** | | | |
| --- | --- | --- | --- |
| **1.** | **Search Question** | RQ1 What are the different regulatory approaches governing wild animal markets, exotic animals, traditional medicine, and exotic pets described in the published literature | |
| **Main concepts** | | | |
| **2.** | **Concepts** | 1. Regulatory approaches 2. Wild animal markets | |
| **3.** | **Search Terms** | | |
| **AND** | **Concept 1** | | **Concept 2** |
|  | Regulatory approaches | | Wild animal markets, traditional medicine, and exotic pets |
| OR | ((Accountability OR  Agreement* OR  Ban* OR  Convention* OR  Governance OR  Guideline* OR  Law* OR  Legal OR  Monitor* OR  Polic* OR  Regime* OR  Regulat* OR  Rule* OR  Standard* OR  Surveillance OR  Treat*) adj1 Trade).ab,ti.  OR  (Legislation OR  Conservation).ab,ti. | | (Exotic animal* OR  Exotic Pet* OR  Traditional Medicine).ab,ti.  OR  ((Bushmeat OR  Illegal OR  Live OR  Meat OR  Wet OR  Wild OR  Wildlife) adj2 Market*).ab,ti.  OR  (Bushmeat trade OR  Bushmeat market).ab,ti. |

| **Search Question** | | | | |
| --- | --- | --- | --- | --- |
| **1.** | **Search Question** | RQ2 What are the different regulatory approaches governing importation of wild animals across international borders described in the published literature? | | |
| **Main concepts** | | | | |
| **2.** | **Concepts** | 1. Regulatory approaches 2. Importation 3. Wild animals 4. International Borders | | |
| **3.** | **Search Terms** | | | |
| **AND** | **Concept 1** | **Concept 2** | **Concept 3** | **Concept 4** |
|  | Regulatory approaches | Importation | Wild animals | International Borders |
| OR | ("Convention on International Trade in Endangered Species of Wild Fauna and Flora" or  "Third-party certificat*" or  "Memorandum of Understanding" or  "Food and Agriculture Organization" or  "World Health Organization" or  "World Food Program" or  "World Organisation for Animal Health").ab,ti.  ((Accountability OR  Agreement* OR  Convention* OR  Governance OR  Guideline* OR  Law* OR  Legal OR  Monitor* OR  Polic* OR  Regime* OR  Regulat* OR  Rule* OR  Standard* OR  Surveillance OR  Treat*) adj1 Trade).ab,ti. | (Export* OR  Importat* OR  Smug* OR  Traffick*).ab,ti. | (Companion Animal* OR  Exotic Animal* OR  Exotic Pet* OR  Farm Animal* OR  Feral Animal* OR  Global wildlife OR  Wildlife OR  Nondomestic Animal* OR  Nondomesticated Animal* OR  Stray Animal* OR  Wild Animal* OR  Wildlife).ab,ti. | (Across OR  Countr* OR  International OR  Regional OR  State OR  Supranational OR  Supra-national).ab,ti. |

| **Search Question** | | | | | |
| --- | --- | --- | --- | --- | --- |
| **1.** | **Search Question** | RQ3 What are the roles of national and global-level institutions in the prevention of local zoonotic spillover and infection transmission described in the published literature on regulatory approaches governing wild animal markets? | | | |
| **Main concepts** | | | | | |
| **2.** | **Concepts** | 1. local, national and global (institutions) 2. prevention 3. zoonotic 4. spillover 5. regulatory approaches | | | |
| **3.** | **Search Terms** | | | | |
| **AND** | **Concept 1** | **Concept 2** | **Concept 3** | **Concept 4** | |
|  | National and Global (institutions) | prevention | zoonotic | Spillover | Regulatory approaches |
| OR | (Cit* OR  Count* OR  Federal OR  Global OR  International OR  Local OR  Metropolitan OR  Municipal OR  National OR  Regional OR  State* OR  Supranational OR  Supra-national).ab,ti. | (Control OR  Emergence* OR  Prevention).ab,ti. | (Zoono* OR  Wildlife).ab,ti. | (Infection* OR  Disease* OR  Spillover OR  Transmission).  ab,ti. | ((Accountability OR  Agreement* OR  Convention* OR  Governance OR  Guideline* OR  Law* OR  Legal OR  Monitor* OR  Polic* OR  Regime* OR  Regulat* OR  Rule* OR  Standard* OR  Surveillance OR  Treat*) adj1 Trade).ab,ti. |
